# Movement Disorders in MOGAD: A Systematic Review

**DOI:** 10.3390/medicina62040693

**Published:** 2026-04-04

**Authors:** Stefania Kalampokini, Antonis Frontistis, Antonis Pilavas, Iraklis Keramidiotis, Marianthi Arnaoutoglou, Vasilios K. Kimiskidis, Effrosyni Koutsouraki

**Affiliations:** 1 1st Department of Neurology, AHEPA University Hospital, Aristotle University of Thessaloniki, 541 24 Thessaloniki, Greece; antonisfront@gmail.com (A.F.); marnaoutoglou@yahoo.com (M.A.); kimiskid@auth.gr (V.K.K.); ekoutsou@auth.gr (E.K.); 2 Medical School, University of Cyprus, Nicosia 1678, Cyprus; antonispilavas@gmail.com; 3 1st Department of Neurosurgery, AHEPA University Hospital, Aristotle University of Thessaloniki, 541 24 Thessaloniki, Greece; dralkis@windowslive.com

**Keywords:** MOGAD, demyelinating disorders, movement disorders, ataxia

## Abstract

*Background and objectives:* Movement disorders are an underrecognized phenomenon in Myelin Oligodendrocyte Glycoprotein-Associated Disease (MOGAD). The aim of this paper was to summarize all movement disorders previously described in MOGAD. *Materials and Methods:* We conducted a systematic literature search in PubMed, Web of Science, and Scopus in English, focusing on patients with MOGAD exhibiting a movement disorder, i.e., ataxia, tremor, dystonia, parkinsonism, chorea, athetosis, myoclonus, ballism, tics, stereotypies, dyskinesia. *Results:* We included 58 studies, with a total of 91 patients with MOGAD and a movement disorder (45.6% male, 54.4% female). Movement disorders had a mean latency of 2.1 years (±6.9, 0–42) after MOGAD onset; however, they could be the presenting feature (in approximately 70% of cases), especially in pediatric patients. Cerebellar ataxia was the most common movement disorder, occurring in 77 patients (84.6%). Tremor, postural and/or kinetic, was the second most common movement disorder (15%). Dystonia was reported in 8.8%, presenting as cervical, or limb dystonia or stereotyped dystonic episodes. Myoclonus and hypokinetic movement disorders were rare. Subcortical (in 60%), brainstem and cerebellar lesions (in 50% respectively) were the most common imaging findings. The most common accompanying symptoms were encephalopathy, fever and headache. Approximately half of the patients made a full recovery, and the other half showed a significant improvement in the movement disorder after immunomodulatory treatment, most commonly steroids. *Conclusions:* The new onset of a movement disorder, especially ataxia, in a young patient should prompt the search for MOGAD or can indicate a relapse in patients with an established diagnosis.

## 1. Introduction

Myelin Oligodendrocyte Glycoprotein-Associated Disease (MOGAD) is an antibody-mediated inflammatory demyelinating disorder of the central nervous system (CNS) with various phenotypes, including optic neuritis, transverse myelitis, acute demyelinating encephalopathy (ADEM), cortical encephalitis, or a combination thereof [1]. MOG is a glycoprotein, produced by oligodendrocytes, on the outer membrane of myelin sheaths expressed within the brain, spinal cord, and optic nerves, which acts as a cell surface receptor or cell adhesion molecule [1]. MOGAD has a trimodal distribution of disease onset, with patients being clustered below 20 years old, between 20 and 45 years old, and over 45 years old, and it is more frequent in children and young adults [2]. Among all ages, there is a great variability in the disease course, with half of the patients having a monophasic disease course and half a relapsing disease course [3]. In patients younger than 20 years old, ADEM is the hallmark presentation, whereas in adult patients, MOGAD is dominated by optic neuritis and transverse myelitis, or additional manifestations such as cortical encephalitis and brainstem involvement [4,5]. MOGAD is characterized by ill-defined, “fluffy” supra- and infratentorial lesions, variable enhancement of the leptomeninges, anterior optic pathway involvement, short or long myelitis lesions, and medullary cone involvement, although central nervous system imaging can be initially normal in up to 10% of cases [6,7].

Movement disorders can be one of the neurological manifestations of inflammatory demyelinating disorders of the CNS or even the presenting feature [8,9]. Some movement disorders occur during an episode of demyelination or can even precede the onset of demyelinating disorders, whereas others can develop later on [10]. In multiple sclerosis (MS), ataxia and tremor, traditionally described in Charcot’s triad, are by far the most common movement disorders, found in up to 80% and 60% of patients, respectively [11], followed by restless legs syndrome, paroxysmal tonic spasms, and hemifacial spasm [8,9]. On the other hand, movement disorders in MOGAD are not so well described, although they are not uncommon, with an incidence ranging from 10% to 50% [12,13,14,15,16,17,18].

To the best of our knowledge, there is no current study concerning the spectrum of movement disorders in MOGAD. In this paper, we aimed to summarize all movement disorders previously described in MOGAD, discuss their clinical and radiological features, their outcome, and the underlying pathophysiology.

## 2. Materials and Methods

A standardized methodology based on PRISMA guidelines was used [19].

### 2.1. Search Strategy and Information Sources

We identified all relevant articles that describe movement disorders in MOGAD. We did a systematic search of articles indexed in Medline (via PubMed), Web of Science, and Scopus from inception to January 2026. The search strategy performed was as follows: (Myelin Oligodendrocyte Glycoprotein Antibody Associated Disease) OR (MOGAD) AND (ataxia) OR (tremor) OR (dystonia) OR (dyskinesia*) OR (parkinsonism)) OR (chorea) OR (athetosis) OR (myoclonus) OR (ballism*) OR (tics) OR (stereotypies) OR (dysmetria) OR (movement disorders) OR (hypokinetic) OR (hyperkinetic). Search results were uploaded into the Rayyan online software (rayyan.ai) and underwent deduplication.

### 2.2. Eligibility Criteria

We included original quantitative studies, case series, and case reports in English that described movement disorders in MOGAD patients as part of their clinical presentation or disease course. Exclusion criteria were articles not written in English, articles without abstract, articles that involved patients with neuromyelitis optica spectrum disorder (NMOSD)/MOGAD overlap or overlap with other inflammatory diseases of the CNS that may act as confounders, articles that involved laboratory animals, articles that did not report on movements disorders in MOGAD patients, and non-original studies/materials (reviews, book chapters, editorials, expert opinions, letters to the editor, conference abstracts).

### 2.3. Screening

Title and abstract screening were performed independently by three reviewers (SK, AP, AF), and conflicts were resolved by discussion until consensus was reached. Article screening was performed on the Rayyan online software (rayyan.ai).

### 2.4. Data Extraction

Data extraction was performed independently by four authors (SK, AP, AF, IK) using a predefined data sheet. From each eligible paper, information extracted was the following: first author, title, year of publication, study objective, population size and characteristics, i.e., age, gender, movement disorder, characters of the movement disorder, movement disorder as presenting symptom, latency of movement disorder after presentation/disease onset, Magnetic resonance imaging (MRI) of the brain and spinal cord findings, other symptoms, treatment, and outcome.

### 2.5. Quality Assessment

The quality of the eligible studies was evaluated using the Joanna Briggs Institute (JBI) critical appraisal tools [20]. The JBI’s Checklist for Analytical Cross-sectional Studies, Case-control studies, Case series, and Case reports critically appraises the methodological quality of a study and determines the possibility of bias in its design, conduct, and analysis using 8, 10, 10, and 8 items, respectively. Each study was categorized as high, moderate, or low quality based on the total score. The authors independently calculated the score for each eligible study during data extraction.

### 2.6. Statistical Analysis

The statistical analysis, i.e., frequencies of the above-mentioned variables, was conducted with SPSS version 25.0. Chi-square tests of independence were conducted to examine the association between the age groups < 18 years old and >18 years old and movement disorders, as well as other clinical and imaging findings of the two groups of patients. Moreover, chi-square tests of independence were conducted to examine the association between movement disorders and specific clinical and imaging findings, as well as the association of relapses within five years with clinical and imaging features and treatment.

## 3. Results

**Figure 1:**
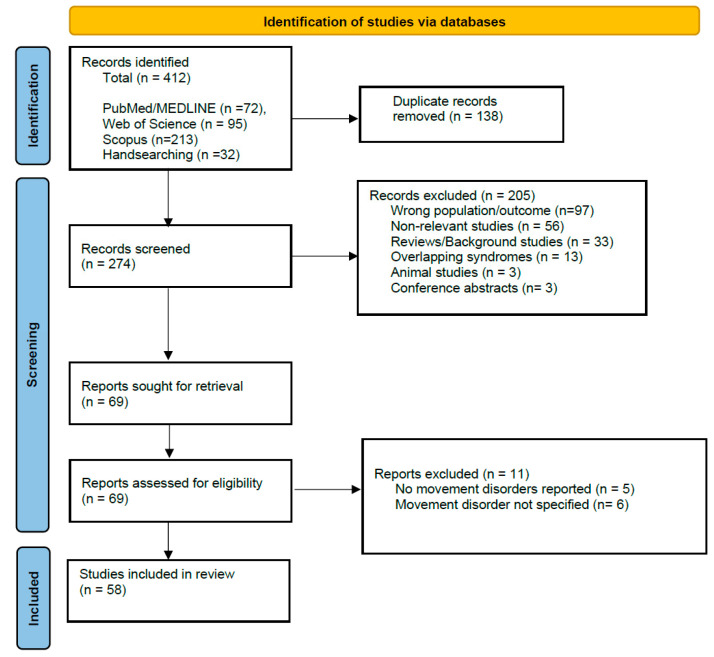
Flow diagram of the included studies.

The initial search from databases yielded 412 studies, of which 138 were duplicates. From the 412 records, after exclusion of studies reporting on non-relevant population or outcome, reviews or background articles, papers on overlapping syndromes, animal studies, and conference abstracts, based on screening of title and abstract, 69 studies were assessed for eligibility, i.e., full text was screened. From those, after excluding studies in which no movement disorder was reported, or the movement disorder was not specified, 58 studies remained for inclusion in the systematic review. Those comprised 20 case-control or cohort studies [12,13,15,16,17,18,21,22,23,24,25,26,27,28,29,30,31,32] and 38 case series or case reports [33,34,35,36,37,38,39,40,41,42,43,44,45,46,47,48,49,50,51,52,53,54,55,56,57,58,59,60,61,62,63,64,65,66,67,68,69,70]. In eight studies, other patient groups were also included, i.e., NMOSD, MS, transverse myelitis, and acute cerebellitis [18,21,22,28,29,31,71,72]. In those cases, data referring only to MOGAD patients were extracted. Notably, the diagnosis of MOGAD was established based on various diagnostic criteria, the most common being those of Jarius et al. [73], Banwell et al. [74], Graus et al. for autoimmune encephalitis [75], or criteria for pediatric immune-mediated CNS demyelinating disorders [76]. In the vast majority of studies, MOG antibodies were measured in serum and, when available, in cerebrospinal fluid. The most common methods of antibody measurement were cell-based assays, live or fixed, when reported. The majority of the included studies (51 studies) were of high quality (7 or higher out of 8 for case reports, 8 or higher out of 10 for case-control studies and case series, 8 or higher out of 11 for cohort studies), and a few (7 studies) were of moderate quality (5–6 out of 8, 6–7 out of 10). The flow chart of the included studies can be seen in Figure 1, and their characteristics can be seen in Supplementary Tables S1 and S2. The demographic, clinical, and imaging characteristics of patients can be seen in Table 1.

### 3.1. Demographics-General Characteristics

Ninety-one patients were included in the analysis. Forty-one (45.6%) were male, 49 (54.4%) female, and in one case, gender was not reported. The median age was 15 years with a range of 4 months to 75 years. The median age of disease onset was 11 years, with a range of 4 months to 75 years. The median follow-up of patients was 24 months, ranging from 6 months to 41.5 years.

**Figure 2:**
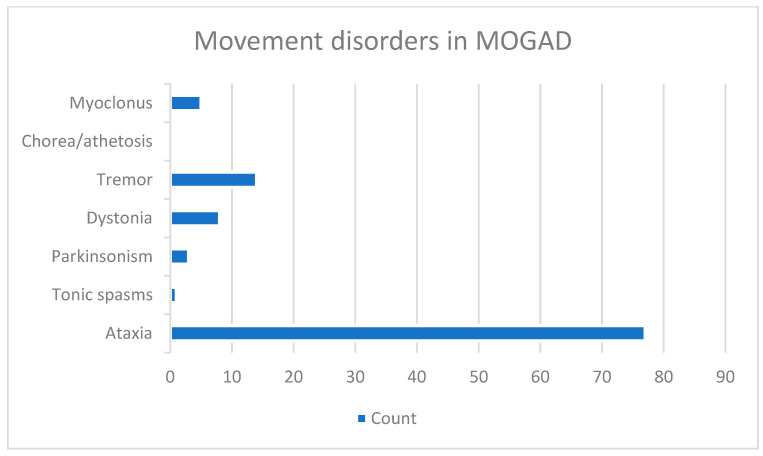
Spectrum of movement disorders reported in MOGAD.

Movement disorders had a mean latency of occurrence of 2.1 years (±6.9) (median 0, range 0–42) after onset of MOGAD. They could be the presenting feature of MOGAD (59 cases, 67.8%), or they could occur after 42 years from disease onset. The spectrum of movement disorders reported in MOGAD can be seen in Figure 2.

### 3.2. Movement Disorders

#### 3.2.1. Ataxia

Ataxia was the most common movement disorder, occurring in 77 patients (84.6%). Thirty-six patients (46.75%) were male and 41 (53.25%) female. Forty patients (51.95%) were younger than 18 years old, and 37 (48.05%) were over 18 years old. Forty-seven patients (64.38%) had ataxia as the presenting symptom of MOGAD. Median age of ataxia presentation was 11 years, with a range of 1.6 to 75 years. The mean latency of ataxia after disease onset was 2.2 ± 7.16 years (median 0, range 0–42). Patients with ataxia were followed up for a median time of 24 months (0–498 months) and had a median number of relapses of 1 (0–22) (mean 1.5 ± 3). Approximately half of those patients (40 patients, 57.97%) relapsed during follow-up; a movement disorder at relapse was observed in 46 patients (73%).

Although the type of ataxia was not defined in most of the included studies, ataxia was, in the vast majority of cases, cerebellar, affecting either the trunk or the limbs [13,15,16,22,24,27]. Twenty-six patients (34.67%) with ataxia also had encephalopathy, i.e., decreased level of consciousness, behavioral and/or memory symptoms as a co-existing symptom, 14 (18.18%) fever, 16 (20.78%) cranial nerve palsy affecting mainly eye movements, 13 (16.88%) motor symptoms, i.e., hemiparesis or paraparesis, 12 (15.58%) sensory or visual symptoms respectively, and 10 (13%) bladder or bowel symptoms.

Concerning MRI in patients with ataxia, 51 patients (67.11%) had subcortical lesions, 40 (52.63%) brainstem lesions, 38 (50%) cerebellar lesions, 17 (22.37%) cortical lesions, and 19 (27.54%) spinal cord lesions. Only two patients had a normal brain and spinal cord MRI. Ataxia, as expected, was found to be associated with cerebellar lesions (Chi-square 8.843, df 1, *p* = 0.003).

#### 3.2.2. Tremor

Tremor was reported in 14 (15.4%) patients in total, presenting as action tremor in most cases [25,55,58]. Only a few patients (3 in total) had a resting component [21,45,67].

#### 3.2.3. Tonic Spasms

Tonic spasms or paroxysmal focal dystonia comprise paroxysmal involuntary sustained muscle contractions of antagonistic muscle groups resulting in abnormal posture, i.e., flexion, extension, or adduction [21]. Notably, those two terms, i.e., paroxysmal focal dystonia and tonic spasms, as well as the term tonic seizures, have been used interchangeably in the literature. Tonic spasms were solely reported in one young adult female patient. The patient had multiple 30 s tonic spasms of the fingers of her right arm.

#### 3.2.4. Dystonia

Dystonia was reported in 8 patients with MOGAD (8.8%). In MOGAD, cervical dystonia has been reported in two preschool children, who generally had a favorable outcome after immunomodulatory treatment (intravenous corticosteroids, immunoglobulins, and rituximab) [29]. There was also a case of severe limb dystonia in both upper extremities in a young boy, which was treated with diazepam, baclofen, and botulinum toxin injections [42]. Repetitive and stereotyped dystonic episodes of the upper and/or lower extremities without any electrographical correlates during the episodes, which required treatment with midazolam and subsided completely after steroid treatment, were described in a young children [53]. Zveik et al. reported another case of a 28-year-old female experiencing repeated right-arm dystonic episodes lasting approximately 30 s, treated initially with carbamazepine (400 mg/day) with a good response and later on with steroids and intravenous immunoglobulin (IVIG), leading to full recovery [64]. Lastly, there was a case of focal dystonia, i.e., blepharospasm in a woman in her early thirties with partial improvement after immunotherapy [55].

#### 3.2.5. Myoclonus

Myoclonus was a rare movement disorder in MOGAD and was mainly focal myoclonus. It has been described in five patients (5.5%), exhibiting jerky movements of the limbs or hemifacial spasm. Opsoclonus myoclonus has been reported in only one case of MOGAD in an adult female patient postpartum (Adhikari, Thuringer, Maali, & Jassam, 2021). There was no case of chorea reported.

#### 3.2.6. Hypokinetic Movement Disorders

Hypokinetic movement disorders were also rare in MOGAD; bradykinesia and cogwheel rigidity were described solely in three cases of two adult patients and one child [51]. The two patients improved on intravenous steroids followed by oral tapering, while the third patient was reported to “do well” on rituximab later on [51].

#### 3.2.7. Mixed Movement Disorders

Although no movement disorder was statistically significantly associated with another, there were rare cases in which the patients exhibited a combined movement disorder. In a few cases, patients presented apart from ataxia, with tremor in 8 cases (10.4%), dystonia in 3 cases (3.9%), parkinsonism in 3 cases (3.9%), and myoclonus in one case. There were no cases of ataxia and tonic spasms.

### 3.3. Imaging Findings

Subcortical lesions were the most common findings in MOGAD patients with movement disorders, reported in 60 patients (66.7%). Brainstem and cerebellar lesions were also common, occurring in 44 patients (48.9%) and 39 patients (43.3%), respectively. Cortical or spinal cord lesions were reported in 25 and 24 patients, respectively (27.8% and 28.9%, respectively). Normal brain and spinal cord MRI were reported in solely 3 cases (3.3%). There was no statistically significant association between movement disorders and imaging findings, except for spinal lesions, i.e., patients with movement disorders had spinal cord lesions less frequently (Chi-square 4.669, df 1, *p* = 0.031).

### 3.4. Accompanying Symptoms

The most common symptoms that accompanied movement disorders in MOGAD were, in descending frequency, encephalopathy in 31 patients (34.8%), fever in 19 patients (20.9%), headache (with or without nausea/vomiting) in 18 (19.8%), motor symptoms, i.e., hemiparesis, paraparesis, tetraparesis in 17 patients (18.7%), cranial nerve deficit, i.e., diplopia, dysphagia in 16 patients, respectively (17.6%), visual symptoms, i.e., blurred vision, decreased visual acuity or sensory symptoms, i.e., numbness, tingling in 14 patients (15.4%), respectively, bladder or bowel problems, i.e., bladder and bowel urgency and incontinence, urinary retention, micturition initiation problems/hesitancy, dysuria in 13 patients (14.3%) and seizures (mostly generalized tonic-clonic) in 6 cases (6.6%). However, there was no statistically significant association of movement disorders with a specific clinical symptom.

### 3.5. Treatment of Relapses and Maintenance Treatment

The vast majority of patients (73, 86.9%) with MOGAD and movement disorders were treated with high-dose intravenous steroids, followed by oral tapering in 64% of the cases (48 patients). The mean duration of tapering was 4.9 ± 9.7 weeks (median 0, 0–48 weeks). IVIG was administered as acute treatment in 26 patients (31%), whereas plasmapheresis (PLEX) was conducted in 10 cases (11.9%). Symptomatic treatment with baclofen, tizanidine, gabapentin, botulinum toxin injections, diazepam, carbamazepine/oxcarbazepine was used in a few cases, in most cases before detection of positive MOG antibodies and initiation of immunotherapy [21,42,57,64]. No patient required tremor-specific treatment, apart from immunotherapy.

Concerning maintenance treatment, this was initiated in the vast majority of cases after one relapse. Oral steroids were the maintenance treatment of choice in 18 patients (23.7%). Thirteen patients (17.1%) were started on mycophenolate, 8 (10.5%) received IVIG monthly or bimonthly as maintenance, 9 patients (11.8%) were started on azathioprine or rituximab, respectively, 2 (2.6%) on methotrexate, tocilizumab, interferons, natalizumab, respectively, and one patient on etanercept, colchicine, infliximab, mitoxantrone, ocrelizumab.

### 3.6. Outcome of Movement Disorders

More than half of the patients (40, 53.3%) made a full recovery, and almost the other half (34, 45.3%) showed a significant improvement in the movement disorder. No improvement was reported in only one case. One adult died at the age of 33, a few months after a severe brainstem attack due to pulmonary infections. Specifically, patients with ataxia, the most common movement disorder, had a full recovery in 52.5% of cases, an improvement or partial recovery in 45.9%, and no improvement in solely 1.6% of cases, after having received immunotherapy (steroids, IVIG, and/or PLEX). Of the 14 patients with tremor, 8 showed improvement, while 6 made a full recovery, in both cases after immunotherapy (most commonly steroids). Concerning the eight patients with dystonia, only two patients made a full recovery, requiring IVIG or PLEX; four showed an improvement, while in two cases, the outcome of the movement disorder was not reported. The five cases of myoclonus, as well as the three cases of parkinsonism had a favorable outcome after immunotherapy, mainly steroids.

### 3.7. Relapses Within 5 Years

For 55 patients, a five-year follow-up was available. Twenty-six patients (47.3%) relapsed within 5 years; the mean relapse number was 0.72 (±1.1) (median 0, range 0–6 relapses). Six patients (12.8%) presented with a movement disorder in relapse, which was ataxia in the vast majority of those. No symptom or imaging finding and no treatment choice was statistically significantly associated with a new relapse within 5 years.

### 3.8. Children (Age < 18 Years Old) vs. Adults (>18 Years Old)

There were 46 patients (50.5%) under the age of 18 and 45 (49.5%) over the age of 18. Patients under 18 years had a movement disorder more frequently as the presenting symptom of MOGAD (85.7% vs. 51.1%, Chi-square 11.918, df 1, *p* = 0.001). There was no statistically significant difference in the type of movement disorder between the two groups. Patients under 18 years also more frequently had encephalopathy as an accompanying symptom (56.8% vs. 13.3%, Chi-square 18.533, df 1, *p* < 0.001) and subcortical lesions on MRI (82.2% vs. 51.1%, Chi-square 9.800, df 1, *p* = 0.002). On the other hand, adults had more frequently motor (28.9% vs. 8.7%, Chi-square 6.106, df 1, *p* = 0.013), sensory (31.1% vs. 0, Chi-square 16.913, df 1, *p* < 0.001) or visual symptoms (26.7% vs. 4.3%, Chi-square 8.704, df 1, *p* = 0.003). There was no other statistically significant difference, neither for clinical nor radiological characteristics, nor in terms of treatment or outcome (including relapses).

## 4. Discussion

In this paper, we summarize movement disorders in MOGAD, which seem to be an underrecognized feature of this inflammatory CNS disorder. Ataxia seems to be the most common disorder in MOGAD. Ataxia was a common presenting symptom of MOGAD, with over 60% of patients exhibiting ataxia as the first symptom [15,22]. Almost one-third of pediatric patients developed ataxia during the course of the disease in a large cohort of pediatric MOGAD patients [77]. Ataxia type was not always specified; however, cerebellar ataxia seems more common than sensory ataxia, the latter occurring as a result of myelitis [25]. The presentation of movement disorders in MOGAD is typically acute or subacute in the context of a relapse; however, there were a few cases with a progressive course of ataxia [33,40,41,69]. Moreover, there were very few cases in which ataxia manifested with another movement disorder at the same time, mostly with limb tremor and rarely with cervical dystonia or parkinsonism [13,25,29,51,58].

In one-third of MOGAD patients, ataxia co-existed with encephalopathy symptoms. Ataxia in MOGAD is often part of a broader encephalopathy-related or ADEM phenotype, such as fever, headache, vomiting, seizures, loss of consciousness, behavioral disorders, or memory complaints, especially in pediatric patients [13,17,24,26,27,29,32]. Indeed, anti-MOG antibodies were more frequent than other neuronal surface antibodies (e.g., anti-GABAb-R, anti-GlyR) in children and young adults with ADEM-like or encephalitic presentations in a recent Brazilian cohort study [23]. Many of those cases are frequently misdiagnosed as MS or Bickerstaff’s brainstem encephalitis [25]. Ataxia in MOGAD can have various neuroimaging correlates, mainly involving the thalamus, brainstem, basal ganglia, cerebellum, and, less frequently, cortical or spinal lesions (cervical or thoracic), usually as part of a multifocal involvement [17,18,22,30]. Indeed, sensory ataxia in MOGAD, resulting from myelitis, has been rarely reported [12,14,18]. MOGAD with brainstem involvement and ataxia as a prevalent sign may represent a distinct MOGAD phenotype [25]. In very young patients, ataxia was associated with a “leucodystrophy-like” phenotype with corresponding imaging findings [13,26]. Notably, apart from the clear correlation of ataxia with cerebellar lesions, over half of MOGAD patients with ataxia had subcortical and/or brainstem lesions, highlighting that ataxia in MOGAD can arise from affection of areas outside the cerebellum such as the thalamus or the internal capsule, the dentato-rubro-thalamo-cortical tract in its way from the midbrain up to the thalamus and motor cortex, or cortico-ponto-cerebellar fibers in the pons [78,79].

Concerning other movement disorders, tremor was the second most common movement disorder, occurring in approximately 15% of patients. Tremor was reported in both adult [21,25,44,51,55,67,68] and pediatric [13,45,52,58,71] patients with MOGAD. Tremor in MOGAD was postural and/or kinetic in nature, mostly involving the hands [21,25,55,58]. The affection of cerebellar pathways, i.e., spinocerebellar tracts seem to be involved in tremor generation in inflammatory demyelinating CNS lesions [80]. There was one reported case of an adult patient exhibiting severe high-frequency “Holmes-like” upper limb tremor, i.e., a combination of rest, posture, and action tremor [67]. Hyperkinetic movements in MOGAD patients are rather a rarity, with five patients exhibiting focal or segmental myoclonus, affecting a limb or the face [23,44,52,66]. Spinal myoclonus seems to be more frequent in NMOSD than MOGAD, apparently due to the frequent occurrence of LETM in the first [21]. No patient exhibiting chorea or athetosis was reported in MOGAD.

Dystonia in MOGAD, observed in less than 10% of cases, and mostly in children [23,29,42,53,57,64] could either present as cervical dystonia [29], limb dystonia [42,57] or facial dystonia, i.e., blepharospasm or orofacial [42,55]. Repetitive and stereotyped dystonic episodes involving the muscles of the upper and lower extremities and/or face have been reported in three cases [53,57,64]. Paroxysmal dystonia has been associated with pain in a few cases in both MOGAD and NMOSD [21]. Hypokinetic movement disorders are also very rare in MOGAD; bradykinesia and cogwheel rigidity were described solely in three cases of two adult patients and one child [51].

In contrast to NMOSD, where the most common movement disorder is tonic spasms of the limbs occurring in almost one fourth of NMOSD patients [81], in MOGAD, tonic spasms are very rare. Indeed, in MOGAD, they have been reported solely in one case [64]. In this case, they occurred in an upper extremity, in contrast to NMOSD, where, in the vast majority of cases, the lower extremities were affected [9]. Moreover, in the case of MOGAD, no spinal cord lesion was detected; on the contrary, the patient had multifocal periventricular and subcortical lesions, corpus callosum involvement with enhancement in the centrum semiovale and corona radiata contralateral to the upper limb exhibiting the tonic spasms [64]. Triggers of tonic spasms include movement, tactile stimulation, nighttime, emotional stress, cold, and bowel movements [82,83]. Tonic spasms have been described after demyelinating lesions of the cerebellum, cerebral peduncles, thalamus, subthalamic nucleus, capsula interna, and basal ganglia, while the spinal cord is the most common lesion site, where the motor (and sensory) fibers are situated close to each other [21,84,85,86]. The affection of the pyramidal and parapyramidal tracts in the spinal cord is presumed to be the origin of tonic spasms [87,88]. The fact that tonic spasms are more prevalent in NMOSD, especially seropositive NMOSD [81], than MOGAD, is likely related to the more extensive inflammation with involvement of motor and spinocerebellar tracts in NMOSD with LETM [21]. On the other hand, MOGAD is a primary demyelinating disorder with a higher remyelination rate [21]. Another reason for tonic spasms being more common in NMOSD than MOGAD could be the higher incidence of cervical lesions in NMOSD compared to conus affection in MOGAD [10]. Therefore, tonic spasms can be used as a clinical key finding that could help differentiate between NMOSD and MOGAD in the initial diagnostic phase.

Concerning brain imaging in MOGAD patients with movement disorders, MRI findings can vary from a normal MRI to large, fluffy lesions [1,3]. Brainstem and cerebellar involvement were common in almost 50% of the patients, respectively, and were frequently found as part of a multifocal CNS attack [18,22]. Pons, medulla oblongata, and middle cerebellar peduncles were the most frequently involved regions in MOGAD with brainstem involvement [18,22,25]. Diffuse lesions in these areas seem to be more frequent in MOGAD than MS or AQP4-IgG-NMOSD [18,22]. Subcortical white matter lesions seem to be common in MOGAD patients, especially frontally, while deep gray matter structures such as the thalamus can also be affected [24]. Another diagnostic clue in MOGAD is the leptomeningeal enhancement, which can occur in up to 40% of patients, and can differentiate MOGAD from NMOSD and MS [15]. Resolution of lesions is often observed in MOGAD and can occur in up to 70% of patients [22]. Initial CNS imaging may be normal in up to one fourth of patients [15]; thus, an acute presentation of a movement disorder without an imaging correlate should include MOGAD in the differential diagnosis.

Concerning different age groups, pediatric patients or patients younger than 18 years had a movement disorder more frequently in the context of a relapse, as a presenting symptom of the disease. The movement disorder was also frequently accompanied by encephalopathy symptoms in our analysis, i.e., decreased level of consciousness, behavioral and/or memory symptoms, which are a common manifestation in younger MOGAD patients. On brain imaging, subcortical lesions were the most common finding in the younger age group with MOGAD. This provides another new observation, that a movement disorder is a characteristic of MOGAD relapses in pediatric or young patients. Although MOG antibody-associated pure cerebellar ataxia with normal CNS imaging is rare, it may constitute a distinct phenotype meriting inclusion in the differential diagnosis of acute ataxia, especially in childhood [29].

Although proving a causal relationship between the demyelinating disorder and the movement disorder is challenging, the acute onset, the time relationship with the clinical event, an imaging correlate on MRI, and response to immunomodulatory treatment strongly suggest causality. On the other hand, the absence of a demyelinating lesion on imaging does not rule out a causal relationship, as the lesion can affect the extrapyramidal pathway at multiple levels or may even be undetectable with standard MRI, or the movement disorder may be the consequence of lesion-induced disturbed neuronal plasticity [8]. Newer metrics of MRI analysis, including T1-weighted hypointense lesions, CNS atrophy measures, magnetization transfer imaging, magnetic resonance spectroscopy, and diffusion tensor imaging, are able to capture a more global image of the range of tissue alterations caused by inflammation and neurodegeneration [89].

Movement disorders, including ataxia, dystonia, tremor, and myoclonus, had a favorable outcome after immunotherapy, with half of the patients making a full recovery, and the other half showing a significant improvement in the movement disorder. As specific treatments for ataxia are lacking, in most MOGAD cases, significant symptom improvement was reported after immunomodulatory treatment [12,14,22]. For acute attacks presenting with ataxia, treatment comprised steroids and/or immunoglobulins and/or plasma exchange, which were effective in most cases. Long-term immunotherapy was most commonly introduced after a relapse and included oral steroids, azathioprine, mycophenolate mofetil, rituximab, or IVIG. Another important issue was that the majority of patients (approximately 70%) who had ataxia during their first relapse had a movement disorder in their following relapse, and this was again, mostly, ataxia. Maintenance therapy, i.e., low-dose oral steroids, mycophenolate, monthly or bimonthly IVIG, azathioprine, or rituximab were used to reduce relapse risk in relapsing patients, i.e., were introduced mostly after a first relapse event [16]. Tapering of oral prednisolone over at least 6 months post-attack, especially in seropositive children, seems a reasonable strategy in MOGAD [16]. In immune-mediated movement disorders, early diagnosis and treatment, as well as escalation of treatment if the patient is not responding to initial treatment, are crucial to minimize the severity of the disease and relapse [90].

Movement disorders as sequelae are reported to occur in up to 25% of pediatric patients with MOGAD in a large cohort study from China [16]. MOGAD is not always benign, 17% of adults have moderate or severe long-term disability, and 42% of pediatric patients have neurological sequelae, i.e., cognitive, behavioral, and/or motor sequelae [12]. Relapses are common, both in children and adults, but particularly in the latter [12,27]. While the diagnostic utility of MOG-IgG testing in serum with live cell-based assays is well established, the role of repeat testing or regular re-testing in patients with MOGAD is less clear [91]. Persistent seropositivity has been associated with a higher rate of relapse that varies substantially from 24 to 88% in the literature, likely reflecting heterogeneity in study design, patient demographics, assay measurements used, and test timing [3,92,93]. In any case, the pragmatic approach of repeating serum testing for anti-MOG antibodies 6–12 months after disease onset to assess for antibody persistence is in accordance with the literature [91]. However, therapeutic decision-making for patients with MOGAD should not be based on MOG-antibody titer alone [30].

A limitation of this review is the various methods used to assess the movement disorders in the different studies, such as regular neurological examination and movement disorder-focused exam. Another issue is that some movement disorders may be mislabeled, which is often the case for paroxysmal dystonia, tonic spasms, cramps, restless legs syndrome, or paresthesia [21]. For example, the terms “tonic spasm” and “paroxysmal dystonia” were frequently used interchangeably in the literature [82]. ‘’Dyskinesia’’ was another broad term comprising involuntary, uncontrolled muscle movements, affecting the face, limbs, or trunk, that was used by a few studies without any further description of movement disorders. Studies using this term were isolated and were not included in our analysis. Moreover, the term ‘’gait instability’’ was occasionally used interchangeably with gait ataxia, causing confusion in certain cases. Classification of ataxia into cerebellar or sensory was also not possible in most cases due to insufficient data. Therefore, a clear classification system for movement disorders in MOGAD is warranted to improve their clinical recognition. Moreover, the diagnosis of MOGAD in the different studies was based on various diagnostic criteria [73,74,76], while many case reports did not mention which criteria the diagnosis was based on. In those case reports, the positivity of MOG antibodies was interpreted as clinically meaningful, due to relevant clinical and imaging findings, based on previous opinion papers or guidelines [75,76]. Although cell-based assays were common, the method of detection of MOG antibodies was also not mentioned in many studies. Notably, in some studies, serum was tested retrospectively for anti-MOG antibodies after an interval of many years [24,41] or the titer was low positive [44,48]. Lastly, another limitation of our review is the synthesis of data derived largely from case series or case reports and case-control or cohort studies, which can be challenging, as these data are inherently heterogeneous, non-independent, and subject to publication bias. Therefore, our findings and reported associations, although suggestive, should be interpreted as exploratory, rather than conclusive.

## 5. Conclusions

The new onset of a movement disorder in a young patient should prompt the search for an inflammatory CNS disease such as MOGAD by obtaining a brain and spinal MRI. In patients with an established diagnosis of inflammatory CNS disorder, the development of a movement disorder can indicate a new relapse and should prompt new imaging, acute relapse management, and consideration of maintenance treatment escalation if indicated [9]. Indeed, the vast majority of movement disorders occurred in the context of a relapse or within a well-defined period after a relapse, i.e., a mean time of 24 months. As the combination of movement disorders and especially ataxia with encephalopathy symptoms seems to be common in MOGAD, anti-MOG should be included in autoimmune encephalitis screening panels, especially in children and patients with ADEM-like or encephalitic presentations [15,23].

Recognizing and treating movement disorders is important due to their impact on quality of life and association with disease activity [21,94,95]. Paroxysmal symptoms are usually underrecognized and under-treated [95]. Movement disorders in MOGAD add to other motor disabilities of patients and can interfere with ambulation or sleep; less commonly can impair skin hygiene, trigger falls, and have a negative impact on activities of everyday living [21]. These findings may help further characterize the clinical manifestations of MOGAD and assist in reaching a diagnosis or including MOGAD in the list of differential diagnoses in the early diagnostic stages.

## Figures and Tables

**Table 1:** Demographics and clinical characteristics of patients with MOGAD and movement disorders included in the review (total N = 91), * in parentheses, number of available cases, valid percentages reported.

Variables		N (%) or Mean (±SD)/Median (Range)
Gender	male female n/a	41 (45.6%) 49 (54.4%) 1 (1%)
Age (years)		15 (0.5–75)
Age at onset (years)		11 (0.5–75)
Movement disorders	ataxia	77 (84.6%)
	tremor	14 (15.4%)
	dystonia	8 (8.8%)
	myoclonus	5 (5.5%)
	parkinsonism	3 (3.3%)
	tonic spasms	1 (1.1%)
Movement disorder as the presenting symptom	yes no	59 (67.8%) 28 (32.2%)
Latency of movement disorder after MOGAD onset (years)		0 (0–42)
Symptoms accompanying movement disorders	encephalopathy (decreased level of consciousness, behavioral and/or memory symptoms)	31 (34.8%)
	fever	19 (20.9%)
	headache	18 (19.8%)
	motor symptoms (hemiparesis, paraparesis)	17 (18.7%)
	cranial nerve palsy	16 (17.6%)
	visual symptoms (blurred vision, decreased visual acuity)	14 (15.4%)
	sensory symptoms (numbness, tingling)	14 (15.4%)
	bladder/bowel disturbance	13 (14.3%)
	seizures	6 (6.6%)
Imaging findings	cortical lesions	25 (27.8%)
	subcortical lesions	60 (66.7%)
	brainstem lesions	44 (48.9%)
	cerebellar lesions	39 (43.3%)
	spinal cord lesions	24 (28.9%)
Acute Treatment (N = 84) *	high-dose intravenous steroids	73 (86.9%)
	IVIG	26 (31%)
	PLEX	10 (11.9%)
	other (diazepam, midazolam, baclofen, botulinum toxin injections, carbamazepine)	4 (4.8%)
Maintenance treatment (N = 76) *	oral steroids	18 (23.7%)
	mycophenolate mofetil	13 (17.1%)
	rituximab	9 (11.8%)
	azathioprine	9 (11.8%)
	IVIG	8 (10.5%)
	οther (methotrexate, tocilizumab, interferons, natalizumab, etanercept, colchicine, infliximab, mitoxantrone, ocrelizumab)	13 (17.1%)
Outcome of movement disorder (N = 75) *	full recovery	40 (53.3%)
	improvement	34 (45.3%)
	no improvement	1 (1.3%)

## Data Availability

Data sharing is not applicable. No new data were created or analyzed in this study.

## Supplementary Materials

The following supporting information can be downloaded at: https://www.mdpi.com/article/10.3390/medicina62040693/s1, Supplementary Table S1: Cohort and case-control studies included in the systematic review, describing MOGAD patients with movement disorders; Supplementary Table S2: Case reports of patients with MOGAD and movement disorders.

## References

[1] Ambrosius W., Michalak S., Kozubski W., Kalinowska A. (2020). Myelin Oligodendrocyte Glycoprotein Antibody-Associated Disease: Current Insights into the Disease Pathophysiology, Diagnosis and Management. Int. J. Mol. Sci..

[2] Jurynczyk M., Messina S., Woodhall M.R., Raza N., Everett R., Roca-Fernandez A., Tackley G., Hamid S., Sheard A., Reynolds G. (2017). Clinical presentation and prognosis in MOG-antibody disease: A UK study. Brain J. Neurol..

[3] Sechi E. (2024). NMOSD and MOGAD. Continuum.

[4] Hor J.Y., Fujihara K. (2023). Epidemiology of myelin oligodendrocyte glycoprotein antibody-associated disease: A review of prevalence and incidence worldwide. Front. Neurol..

[5] Ciampi E. (2025). MOGAD: A Shifting Landscape-From Pathogenesis to Personalised Management, Global Perspectives and Latin American Insights. Biomedicines.

[6] Sechi E., Cacciaguerra L., Chen J.J., Mariotto S., Fadda G., Dinoto A., Lopez-Chiriboga A.S., Pittock S.J., Flanagan E.P. (2022). Myelin Oligodendrocyte Glycoprotein Antibody-Associated Disease (MOGAD): A Review of Clinical and MRI Features, Diagnosis, and Management. Front. Neurol..

[7] Marignier R., Hacohen Y., Cobo-Calvo A., Pröbstel A.K., Aktas O., Alexopoulos H., Amato M.P., Asgari N., Banwell B., Bennett J. (2021). Myelin-oligodendrocyte glycoprotein antibody-associated disease. Lancet Neurol..

[8] Mehanna R., Jankovic J. (2013). Movement disorders in multiple sclerosis and other demyelinating diseases. J. Neurol. Sci..

[9] Abboud H., Yu X.X., Knusel K., Fernandez H.H., Cohen J.A. (2019). Movement disorders in early MS and related diseases: A prospective observational study. Neurol. Clin. Pract..

[10] Singh R., Pandey S. (2022). Movement Disorder in Demyelinating Disease: Tracing the Charcot’s Foot Print. Ann. Indian Acad. Neurol..

[11] Ghosh R., Roy D., Dubey S., Das S., Benito-León J. (2022). Movement Disorders in Multiple Sclerosis: An Update. Tremor Other Hyperkinet. Mov..

[12] Boudjani H., Fadda G., Dufort G., Antel J., Giacomini P., Levesque-Roy M., Oskoui M., Duquette P., Prat A., Girard M. (2023). Clinical course, imaging, and pathological features of 45 adult and pediatric cases of myelin oligodendrocyte glycoprotein antibody-associated disease. Mult. Scler. Relat. Disord..

[13] Hacohen Y., Rossor T., Mankad K., Chong W., Lux A., Wassmer E., Lim M., Barkhof F., Ciccarelli O., Hemingway C. (2018). ‘Leukodystrophy-like’ phenotype in children with myelin oligodendrocyte glycoprotein antibody-associated disease. Dev. Med. Child. Neurol..

[14] Jarius S., Ruprecht K., Kleiter I., Borisow N., Asgari N., Pitarokoili K., Pache F., Stich O., Beume L.A., Hümmert M.W. (2016). MOG-IgG in NMO and related disorders: A multicenter study of 50 patients. Part 2: Epidemiology, clinical presentation, radiological and laboratory features, treatment responses, and long-term outcome. J. Neuroinflamm..

[15] Kim N.N., Champsas D., Eyre M., Abdel-Mannan O., Lee V., Skippen A., Chitre M.V., Forsyth R., Hemingway C., Kneen R. (2024). Pediatric MOG-Ab-Associated Encephalitis: Supporting Early Recognition and Treatment. Neurol. Neuroimmunol. Neuroinflamm..

[16] Li L., Liu W., Cai Q., Liu Y., Hu W., Zuo Z., Ma Q., He S., Jin K. (2023). Leptomeningeal enhancement of myelin oligodendrocyte glycoprotein antibody-associated encephalitis: Uncovering novel markers on contrast-enhanced fluid-attenuated inversion recovery images. Front. Immunol..

[17] Wang Y., Guo X., Zhang L., Hua Y., Jing M., Hu X., Fan X., Sun M., Liu Y., Wang J. (2024). Clinical characteristics analysis of 24 cases of pediatric MOG antibody-associated diseases. Mult. Scler. Relat. Disord..

[18] Xu Q., Yang X., Qiu Z., Li D., Wang H., Ye H., Jiao L., Zhang J., Di L., Lei P. (2023). Clinical features of MOGAD with brainstem involvement in the initial attack versus NMOSD and MS. Mult. Scler. Relat. Disord..

[19] Tricco A.C., Lillie E., Zarin W., O’Brien K.K., Colquhoun H., Levac D., Moher D., Peters M.D.J., Horsley T., Weeks L. (2018). PRISMA Extension for Scoping Reviews (PRISMA-ScR): Checklist and Explanation. Ann. Intern. Med..

[20] Ma L.L., Wang Y.Y., Yang Z.H., Huang D., Weng H., Zeng X.T. (2020). Methodological quality (risk of bias) assessment tools for primary and secondary medical studies: What are they and which is better?. Mil. Med. Res..

[21] Abboud H., Sun R., Modak N., Elkasaby M., Wang A., Levy M. (2024). Spinal movement disorders in NMOSD, MOGAD, and idiopathic transverse myelitis: A prospective observational study. J. Neurol..

[22] Banks S.A., Morris P.P., Chen J.J., Pittock S.J., Sechi E., Kunchok A., Tillema J.M., Fryer J.P., Weinshenker B.G., Krecke K.N. (2020). Brainstem and cerebellar involvement in MOG-IgG-associated disorder versus aquaporin-4-IgG and MS. J. Neurol. Neurosurg. Psychiatry.

[23] de Freitas Dias B., Toso F.F., Barreto M., Dellavance A., Thomaz R.B., Kowacs P.A., Teive H., Spitz M., Juliano A.F.B., Rocha L.J.A. (2024). Frequency of anti-MOG antibodies in serum and CSF of patients with possible autoimmune encephalitis: Results from a Brazilian multicentric study. Mult. Scler. Relat. Disord..

[24] George E., Russ J.B., Validrighi A., Early H., Mamlouk M.D., Glenn O.A., Francisco C.M., Waubant E., Lindan C., Li Y. (2024). Clinical and Imaging Findings in Children with Myelin Oligodendrocyte Glycoprotein Antibody Associated Disease (MOGAD): From Presentation to Relapse. AJNR Am. J. Neuroradiol..

[25] Jarius S., Kleiter I., Ruprecht K., Asgari N., Pitarokoili K., Borisow N., Hümmert M.W., Trebst C., Pache F., Winkelmann A. (2016). MOG-IgG in NMO and related disorders: A multicenter study of 50 patients. Part 3: Brainstem involvement—frequency, presentation and outcome. J. Neuroinflamm..

[26] Jiang Y., Tan C., Li X., Jiang L., Hong S., Yuan P., Zheng H., Fan X., Han W. (2023). Clinical features of the first attack with leukodystrophy-like phenotype in children with myelin oligodendrocyte glycoprotein antibody-associated disorders. Int. J. Dev. Neurosci..

[27] Kang Q., Liao H., Yang L., Fang H., Ning Z., Liao C., Gan S., Wu L. (2023). Clinical analysis of 173 pediatric patients with antibody-mediated autoimmune diseases of the central nervous system: A single-center cohort study. Front. Immunol..

[28] Liu H., Zhang X., Chen W., Xu Y., Lin X., Lin A. (2024). The relationship between plasma prolactin levels and clinical manifestations with neuromyelitis optica spectrum disorders. Neurol. Sci. Off. J. Ital. Neurol. Soc. Ital. Soc. Clin. Neurophysiol..

[29] Quack L., Glatter S., Wegener-Panzer A., Cleaveland R., Bertolini A., Endmayr V., Seidl R., Breu M., Wendel E., Schimmel M. (2023). Autoantibody status, neuroradiological and clinical findings in children with acute cerebellitis. Eur. J. Paediatr. Neurol..

[30] Serin H.M., Yilmaz S., Simsek E., Kanmaz S., Eraslan C., Aktan G., Tekgul H., Gokben S. (2021). Clinical spectrum, treatment and outcome of myelin oligodendrocyte glycoprotein (MOG) antibody-associated disease in children: A tertiary care experience. Acta Neurol. Belg..

[31] Siritho S., Sato D.K., Kaneko K., Fujihara K., Prayoonwiwat N. (2016). The clinical spectrum associated with myelin oligodendrocyte glycoprotein antibodies (anti-MOG-Ab) in Thai patients. Mult. Scler..

[32] Song X., Ma J. (2022). Clinical characteristics of myelin-oligodendrocyte glycoprotein antibody-positive pediatric autoimmune encephalitis without demyelination: A case series. Front. Immunol..

[33] Alshamrani F., Alnajashi H., Shosha E., Casserly C., Morrow S.A. (2020). Case Series: Myelin Oligodendrocyte Glycoprotein-Immunoglobulin G-Related Disease Spectrum. Front. Neurol..

[34] Aubart M., Roux C.J., Durrleman C., Gins C., Hully M., Kossorotoff M., Gitiaux C., Levy R., Moulin F., Debray A. (2022). Neuroinflammatory Disease following Severe Acute Respiratory Syndrome Coronavirus 2 Infection in Children. J. Pediatr..

[35] Bogdan T., El Ghannudi S., Demuth S., Kremer L., De Seze J., Bigaut K. (2022). Reverse Takotsubo cardiomyopathy as a complication of MOG-antibody-associated disease (MOGAD)? A case report. Rev. Neurol..

[36] Daems F., Derdelinckx J., Ceyssens S., Vanden Bossche S., Reynders T., Willekens B. (2023). Improved detection of MOG antibody-associated transverse myelitis with 18F-FDG-PET: A case report. Acta Neurol. Belg..

[37] El Jammal T., Jamilloux Y., Gerfaud-Valentin M., Richard-Colmant G., Weber E., Bert A., Androdias G., Sève P. (2021). Challenging Mimickers in the Diagnosis of Sarcoidosis: A Case Study. Diagnostics.

[38] Fujimori J., Takahashi T., Matsumoto Y., Fujihara K., Takai Y., Misu T., Nakashima I. (2019). Two Japanese cases of anti-MOG antibody-associated encephalitis that mimicked neuro-Behçet’s disease. J. Neuroimmunol..

[39] García-Estrada C., Gomez-Figueroa E., Morelos-Cisneros J.P., Deras-Martinez A. (2022). Myelin oligodendrocyte glycoprotein antibody-associated disease presenting as recurrent acute disseminated encephalomyelitis: Case report of the youngest Mexican patient in the literature. Clin. Exp. Neuroimmunol..

[40] Gibbons E., Whittam D., Elhadd K., Bhojak M., Rathi N., Avula S., Jacob A., Griffiths M., Huda S. (2022). Progressive myelin oligodendrocyte glycoprotein-associated demyelination mimicking leukodystrophy. Mult. Scler..

[41] Gil-Perotin S., Castillo-Villalba J., Carreres-Polo J., Navarré-Gimeno A., Mallada-Frechín J., Pérez-Miralles F., Gascón F., Alcalá-Vicente C., Cubas-Nuñez L., Casanova-Estruch B. (2018). Progressive Demyelination in the Presence of Serum Myelin Oligodendrocyte Glycoprotein-IgG: A Case Report. Front. Neurol..

[42] Khan T.R., Waugh J.L., Wang C. (2021). Anti-Myelin Oligodendrocyte Glycoprotein (MOG) antibody disease presenting with severe dystonia. Neuroimmunol. Rep..

[43] Krett J.D., Jarvis S.E., Alikhani K. (2021). Myelin Oligodendrocyte Glycoprotein Antibody-Associated Myelitis Presenting with Headache. Can. J. Neurol. Sci. J. Can. Des. Sci. Neurol..

[44] Adhikari S., Thuringer A., Maali L., Jassam Y. (2021). Opsoclonus myoclonus syndrome in a postpartum period. Mult. Scler. Relat. Disord..

[45] Lopes T.A., Cordeiro C., Goncalves R., Pais R.P., Palavra F. (2023). Atypical Cerebellar Involvement in MOG Antibody-Associated Disease (MOGAD) in Early Childhood. Sinapse.

[46] Maniscalco G.T., Allegorico L., Alfieri G., Napolitano M., Ranieri A., Renna R., Servillo G., Pezzella M., Capone E., Altomare L. (2021). Anti-MOG-associated demyelinating disorders: Two sides of the same coin. Neurol. Sci. Off. J. Ital. Neurol. Soc. Ital. Soc. Clin. Neurophysiol..

[47] Mariotto S., Monaco S., Peschl P., Coledan I., Mazzi R., Höftberger R., Reindl M., Ferrari S. (2017). MOG antibody seropositivity in a patient with encephalitis: Beyond the classical syndrome. BMC Neurol..

[48] Mayuzumi Y., Kitazawa Y., Kunimatsu T. (2023). Relapse of myelin oligodendrocyte glycoprotein antibody-associated demyelinating disease in an elderly patient after long-term remission. Acta Neurol. Belg..

[49] Nakano H., Yamaguchi K., Hama N., Matsumoto Y., Shinohara M., Ide H. (2023). Relapsing Anti-MOG Antibody-associated Disease following COVID-19 Vaccination: A Rare Case Report and Review of the Literature. Intern. Med..

[50] Okubo S., Kakumoto T., Tsujita M., Muramatsu K., Fujiwara S., Hamada M., Satake W., Toda T. (2024). Extremely Longitudinally Extensive Transverse Myelitis in a Patient with Myelin Oligodendrocyte Glycoprotein Antibody-Associated Disease. Cureus.

[51] Poovathingal M.A. (2024). The Varying Faces of MOGAD: A Case Series. Ann. Afr. Med..

[52] Ramanathan S., O’Grady G.L., Malone S., Spooner C.G., Brown D.A., Gill D., Brilot F., Dale R.C. (2019). Isolated seizures during the first episode of relapsing myelin oligodendrocyte glycoprotein antibody-associated demyelination in children. Dev. Med. Child. Neurol..

[53] Sa M., Thornton R., Chong W.K., Kaliakatsos M., Hacohen Y. (2019). Paediatric MOG antibody-associated ADEM with complex movement disorder: A case report. Mult. Scler..

[54] Schirò G., Iacono S., Andolina M., Bianchi A., Ragonese P., Salemi G. (2024). Tocilizumab treatment in MOGAD: A case report and literature review. Neurol. Sci. Off. J. Ital. Neurol. Soc. Ital. Soc. Clin. Neurophysiol..

[55] Seneviratne S.O., Marriott M., Ramanathan S., Yeh W., Brilot-Turville F., Butzkueven H., Monif M. (2022). Failure of alemtuzumab therapy in three patients with MOG antibody associated disease. BMC Neurol..

[56] Smoot K., RN C.C., Cohan S. (2021). Recurrent relapse after 20 years in a patient with MOG antibody disease: A case report. Neuroimmunol. Rep..

[57] Redžek-Mudrinić Τ., Kavecan I., Koprivšek K., Rakić G., Pajić J. (2022). Pediatric acute disseminated encephalomyelitis associated with myelin oligodendrocyte glycoprotein antibodies. Srp. Arh. Celok. Lek..

[58] Thulasirajah S., Pohl D., Davila-Acosta J., Venkateswaran S. (2016). Myelin Oligodendrocyte Glycoprotein-Associated Pediatric Central Nervous System Demyelination: Clinical Course, Neuroimaging Findings, and Response to Therapy. Neuropediatrics.

[59] Vieira J.P., Sequeira J., Brito M.J. (2017). Postinfectious Anti-Myelin Oligodendrocyte Glycoprotein Antibody Positive Optic Neuritis and Myelitis. J. Child. Neurol..

[60] Wang X., Zhao R., Yang H., Liu C., Zhao Q. (2023). Two rare cases of myelin oligodendrocyte glycoprotein antibody-associated disorder in children with leukodystrophy-like imaging findings. BMC Neurol..

[61] Xu M., Ma C., Dong M., Guo C., Yang S., Liu Y., Wang X. (2023). Two case reports and a systematic review of the literature on adult cerebral cortical encephalitis with anti-myelin oligodendrocyte glycoprotein antibody. Front. Immunol..

[62] Yılmaz Ü., Edizer S., Songür Ç Y., Güzin Y., Durak F.S. (2019). Atypical presentation of MOG-related disease: Slowly progressive behavioral and personality changes following a seizure. Mult. Scler. Relat. Disord..

[63] Zehden J., Harish Bindiganavile S., Bhat N., Lee A.G., Avery R., Golnik K.C. (2022). Delayed Diagnosis of Anti-Myelin Oligodendrocyte Glycoprotein One Decade After Presumed Recurrent Acute Disseminated Encephalomyelitis. J. Neuroophthalmol..

[64] Zveik-Lavi O., Berman T.B., Keadan T., Barhum K., Rechtman A., Vaknin-Dembinsky A. (2023). Myelin oligodendrocyte glycoprotein antibody-associated disease presenting with dystonia. Neurol. Clin. Neurosci..

[65] Chakraborty U., Ghosh S., Datta A.K., Chandra A. (2021). Recurrent ataxia and dysarthria in myelin oligodendrocyte glycoprotein antibody-associated disorder. BMJ Case Rep..

[66] Holay Q., Gazzola S., Quesnel L., Faivre A. (2023). Migrating cortical lesion in FLAIR-hyperintense lesions in anti-MOG-associated encephalitis with seizures. J. Neurol. Neurosurg. Psychiatry.

[67] Kumar N., Graven K., Joseph N.I., Johnson J., Fulton S., Hostoffer R., Abboud H. (2020). Case Report: Postvaccination Anti-Myelin Oligodendrocyte Glycoprotein Neuromyelitis Optica Spectrum Disorder: A Case Report and Literature Review of Postvaccination Demyelination. Int. J. MS Care.

[68] Mishan Y., Schwartz D., Elefant D., Gandelman S. (2025). Late-onset MOGAD: A case series and literature review. Neuroimmunol. Rep..

[69] Baddam S., Patel S., Kahlon N., Thiriveedi M. (2025). Unmasking Myelin Oligodendrocyte Glycoprotein Antibody-Associated Disease (MOGAD): CNS Demyelination Triggered by TNF-α Inhibition in a Patient with Ankylosing Spondylitis. Eur. J. Case Rep. Intern. Med..

[70] Sinha N., Prakash P., Ranjan A. (2025). Chronic progressive behavioral changes associated with headache: An atypical presentation of myelin oligodendrocyte glycoprotein (MOG)-associated disease. Turk. J. Neurol..

[71] Xiao J., Zhang S.Q., Chen X., Tang Y., Chen M., Shang K., Deng G., Qin C., Tian D.S. (2022). Comparison of clinical and radiological characteristics in autoimmune GFAP astrocytopathy, MOGAD and AQP4-IgG(+) NMOSD mimicking intracranial infection as the initial manifestation. Mult. Scler. Relat. Disord..

[72] Zhao Q., Gao C., Wang L., Liu C., Sun S., Li B. (2025). Acute ataxia in children: Etiological spectrum and clinical characteristics. Front. Pediatr..

[73] Jarius S., Paul F., Aktas O., Asgari N., Dale R.C., de Seze J., Franciotta D., Fujihara K., Jacob A., Kim H.J. (2018). MOG encephalomyelitis: International recommendations on diagnosis and antibody testing. J. Neuroinflamm..

[74] Banwell B., Bennett J.L., Marignier R., Kim H.J., Brilot F., Flanagan E.P., Ramanathan S., Waters P., Tenembaum S., Graves J.S. (2023). Diagnosis of myelin oligodendrocyte glycoprotein antibody-associated disease: International MOGAD Panel proposed criteria. Lancet Neurol..

[75] Graus F., Titulaer M.J., Balu R., Benseler S., Bien C.G., Cellucci T., Cortese I., Dale R.C., Gelfand J.M., Geschwind M. (2016). A clinical approach to diagnosis of autoimmune encephalitis. Lancet Neurol..

[76] Krupp L.B., Tardieu M., Amato M.P., Banwell B., Chitnis T., Dale R.C., Ghezzi A., Hintzen R., Kornberg A., Pohl D. (2013). International Pediatric Multiple Sclerosis Study Group criteria for pediatric multiple sclerosis and immune-mediated central nervous system demyelinating disorders: Revisions to the 2007 definitions. Mult. Scler..

[77] Li X., Wu W., Hou C., Zeng Y., Wu W., Chen L., Liao Y., Zhu H., Tian Y., Peng B. (2023). Pediatric myelin oligodendrocyte glycoprotein antibody-associated disease in southern China: Analysis of 93 cases. Front. Immunol..

[78] Marsden J.F. (2018). Cerebellar ataxia. Handb. Clin. Neurol..

[79] Pedroso J.L., Vale T.C., Braga-Neto P., Dutra L.A., França M.C., Teive H.A.G., Barsottini O.G.P. (2019). Acute cerebellar ataxia: Differential diagnosis and clinical approach. Arq. Neuro-Psiquiatr..

[80] Shneyder N., Harris M.K., Minagar A. (2011). Movement disorders in patients with multiple sclerosis. Handb. Clin. Neurol..

[81] Bringel L.A.F., Lima P., Rodrigues P.V.F., Cavalcante F., Pinheiro A.R., Saraiva F.M.S., Rodrigues C.P., Gonçalves P.V.S., Melo L., Camelo-Filho A.E. (2025). Movement Disorders in Neuromyelitis Optica Spectrum Disorder: A Systematic Review. Mov. Disord. Clin. Pract..

[82] Abaroa L., Rodríguez-Quiroga S.A., Melamud L., Arakaki T., Garretto N.S., Villa A.M. (2013). Tonic spasms are a common clinical manifestation in patients with neuromyelitis optica. Arq. Neuro-Psiquiatr..

[83] Abboud H., Fernandez H.H., Mealy M.A., Levy M. (2016). Spinal Movement Disorders in Neuromyelitis Optica: An Under-recognized Phenomenon. Mov. Disord. Clin. Pract..

[84] Tranchant C., Bhatia K.P., Marsden C.D. (1995). Movement disorders in multiple sclerosis. Mov. Disord. Off. J. Mov. Disord. Soc..

[85] Usmani N., Bedi G., Lam B.L., Sheremata W.A. (2012). Association between paroxysmal tonic spasms and neuromyelitis optica. Arch. Neurol..

[86] Carnero Contentti E., Leguizamón F., Hryb J.P., Celso J., Pace J.L., Ferrari J., Knorre E., Perassolo M.B. (2016). Neuromyelitis optica: Association with paroxysmal painful tonic spasms. Neurologia.

[87] Abboud H., Macaron G., Yu X.X., Knusel K., Fernandez H.H., Bethoux F. (2019). Defining the spectrum of spasticity-associated involuntary movements. Park. Relat. Disord..

[88] Dressler D., Bhidayasiri R., Bohlega S., Chana P., Chien H.F., Chung T.M., Colosimo C., Ebke M., Fedoroff K., Frank B. (2018). Defining spasticity: A new approach considering current movement disorders terminology and botulinum toxin therapy. J. Neurol..

[89] Zivadinov R., Cox J.L. (2007). Neuroimaging in multiple sclerosis. Int. Rev. Neurobiol..

[90] Mohammad S.S., Dale R.C. (2018). Principles and approaches to the treatment of immune-mediated movement disorders. Eur. J. Paediatr. Neurol..

[91] Budhram A., Flanagan E.P. (2025). Testing for myelin oligodendrocyte glycoprotein antibodies: Who, what, where, when, why, and how. Mult. Scler..

[92] Waters P., Fadda G., Woodhall M., O’Mahony J., Brown R.A., Castro D.A., Longoni G., Irani S.R., Sun B., Yeh E.A. (2020). Serial Anti-Myelin Oligodendrocyte Glycoprotein Antibody Analyses and Outcomes in Children with Demyelinating Syndromes. JAMA Neurol..

[93] López-Chiriboga A.S., Majed M., Fryer J., Dubey D., McKeon A., Flanagan E.P., Jitprapaikulsan J., Kothapalli N., Tillema J.M., Chen J. (2018). Association of MOG-IgG Serostatus with Relapse After Acute Disseminated Encephalomyelitis and Proposed Diagnostic Criteria for MOG-IgG-Associated Disorders. JAMA Neurol..

[94] Candeias da Silva C., Bichuetti D.B., Azevedo Silva S.M.C., Ferraz H.B., Oliveira E.M.L., Borges V. (2018). Movement disorders in multiple sclerosis and neuromyelitis optica: A clinical marker of neurological disability. Park. Relat. Disord..

[95] Lotan I., Bacon T., Kister I., Levy M. (2020). Paroxysmal symptoms in neuromyelitis optica spectrum disorder: Results from an online patient survey. Mult. Scler. Relat. Disord..

