# Supplementary material for: Movement Disorders in MOGAD: A Systematic Review
Source: Medicina (Kaunas). 2026 Apr 4;62(4):693. doi: 10.3390/medicina62040693 (PMC13117712; doi:10.3390/medicina62040693)
Supplement: Supplementary file 1 [file medicina-62-00693-s001.zip › supplementary Table S1 cohort studies MOGAD SK.pdf]

Author	Type of study	Number of MOGAD patients	Number of patients with movement disorders	Gender	Age at onset in years (median, range)	Movement disorders	Imaging findings*	Other symptoms*	Treatment*	Outcome of movement disorder
**Siritho, 2015 [1]**	Retrospective Case Series	6	1	1F	20	Ataxia	Subcortical lesions, Spinal cord (LETM)	Hemiparesis	N/A	N/A
**Jarius, 2016 [2]**	Retrospective, Case Series	50	5	1M, 4F	25 (13-44)	Ataxia (5), Tremor (1)	Cortical lesions (3), Subcortical lesions (2), Brainstem (2), Cerebellum (2), Spinal cord (4) (LETM)	Headache (1), Motor (3), Visual (1), Bladder/Bowel (2)	Steroids (5), PLEX (2)<br><br>Maintenance: AZA (3), MTX (1), RTX (2), MTX (1)	Partial improvement (5)
**Thulasirajah, 2016 [3]**	Case series	5	3	1M, 2F	6 (3-8)	Ataxia (3), Tremor (1)	Subcortical lesions (3), Brainstem (1), Spinal cord (2)	Encephalopathy (1), Headache (1), Cranial nerves (1), Bladder/Bowel symptoms (1)	Steroids (3), IVIG (2)	Complete recovery (2), partial recovery (1)
**Hacohen, 2018 [4]**	Retrospective Observational	31	5	1M, 4F	4 (1.7-6)	Ataxia	Subcortical lesions (4), Brainstem (2), Cerebellum (2)	Encephalopathy (3), Fever (1), Cranial nerves (1)	Steroids (4)<br><br>Maintenance: IFN (2), AZA (2), NTZ (1)	Improvement (2), complete recovery (2), no improvement (1)

**Ramanathan, 2019 [5]**	Case series	4	2	2F	6.5 (3-10)	Myoclonus	Cortical lesions (1), Subcortical lesions (2)	Encephalopathy (1)	Steroids (1)<br><br>Maintenance: MMF (1)	Complete recovery (1), Partial improvement (1)
**Serin, 2020 [6]**	Retrospective case series	9	5	1M, 4F	6 (3-11)	Ataxia	Cortical lesions (1), Subcortical lesions (5), Brainstem (3), Cerebellum (4), Spinal Cord (4) (LETM)	Encephalopathy (4), Headache (2)	Steroids<br><br>Maintenance: IVIG (3), AZA-MTX-MMF-RTX-IFX (1)	Complete recovery
**Alshamrani, 2020 [7]**	Case series	11	2	2F	43.5 (29-58)	Ataxia	Subcortical lesions (1)	Cranial nerves (1), Bladder/Bowel symptoms (1)	IVIG (1)<br><br>Maintenance: MMF (1), AZA (1)	Complete recovery (1), Improvement (1)
**Banks, 2021 [8]**	Retrospective Observational	185	17	N/A	N/A	Ataxia	Brainstem, Cerebellum	Diplopia, Intractable Nausea, Vomiting, Vertigo, Dysarthria, Cranial Nerve Palsy	Steroids, IVIG, PLEX, RTX, AZA	Complete Resolution in 67%*

**Song, 2022 [9]**	Retrospective Case Series	18	2	2F	8.95 (7-10.9)	Ataxia	Cortical lesions (1), Subcortical lesions (1), Brainstem (1), Cerebellum (2)	Encephalopathy (2), Fever (2), Headache (1), Cranial Nerves (1)	Steroids (2), IVIG (2)	Partial recovery
**Xiao, 2022 [10]**	Retrospective Observational, single center	17	1	1M	37	Tremor	Cortical, brainstem lesions	Fever, Headache	Steroids	Complete recovery
**Aubart, 2022 [11]**	Case series	3	2	2M	6.1 (1.6-10.6)	Ataxia	Cortical lesions (1), subcortical lesions (1), Brainstem 2), Cerebellum (2)	Fever (1), Motor (1), Bladder/Bowel (1)	Steroids (1)	Complete recovery (2)
**Severinatne, 2022 [12]**	Case series	3	2	2F	46.5 (34-59)	Ataxia (1), Dystonia (1), Tremor (1)	Cortical lesions (1), Subcortical lesions (1), Spinal cord (1)	Cranial nerves (1), Motor (1), Sensory (1), Visual symptoms (1)	Steroids (2), IVIG (1)<br><br>Maintenance: NTZ (1), OCR (1), MMF (2)	Partial Improveme nt
**Boudjani, 2023 [13]**	Prospective Observational, Cohort design	45	7	N/A	32 (1-71)	Ataxia	Corpus Callosum, Spinal Cord	Vomiting, Cranial Nerve Palsy, Seizures, Diplopia, Sensory Disturbances, Cognitive Deficits, Sphincter Dysfunction	IV Steroids, IVIG, PLEX<br><br>Maintenance: AZA, IVIG, RTX, MMF	Good recovery in 88%, poor recovery in 12%*

**Jiang, 2023 [14]**	Retrospective, Case Series	4	2	2M	2.62 (2-3.25)	Ataxia	Cortical lesions (1), Subcortical lesions (1), Cerebellum (1)	Encephalopathy (2), Fever (1), Headache (1)	Steroids (2), IVIG (1)	Complete recovery
**Kang, 2023 [15]**	Retrospective Observational, Cohort design	48	2	1M, 1F	8 (4-12)	Ataxia	Subcortical lesions	Headache (1), muscle weakness (1)	N/A	N/A
**Li, 2023 [16]**	Retrospective Observational	93	27	N/A	N/A	Ataxia	Basal Ganglia, Subcortical White Matter, Brainstem, Cerebellum, Spinal Cord (LETM)	Seizures, Headache, Fever, Vomiting Visual Deficits, Limb Paralysis, Cranial Nerve Palsy, Speech Disturbance	Steroids, IVIG, PLEX<br><br>Maintenance: RTX, MMF	Sequelae in 25.6%*
**Quack, 2023 [17]**	Retrospective Multicenter Observational	5	4	3M, 1F	4 (2.4-4)	Ataxia (4), Dystonia (2)	Cortical lesions (2), Subcortical lesions (4), Cerebellum (4)	Encephalopathy (1), Fever (2), Headache (1), Hemiparesis (1)	N/A	N/A
**Xu, 2023 [18]**	Retrospective Observational Study	35	7	4M, 3F	28 (19-54)	Ataxia	Subcortical lesions (2), Brainstem (7), Cerebellum (4)	Encephalopathy (1), Headache (1), Seizures (1), Cranial nerves (4), Motor (3), Sensory (2), Visual (4)	Steroids (7)<br><br>Maintenance: MMF (4)	N/A

**Abboud, 2024 [19]**	Prospective Observational, Cohort design	7	3	1M, 2F	41	Dystonia, Tremor	Cortical Lesions, Brainstem, Spinal Cord (cervical, thoracic)	Spasticity	Baclofen, Tizanidine, Botulinum Toxin, Carbamazepine/ Oxcarbazepine, Gabapentin	Complete Recovery (19.5%), Partial Improvement (37%), Persistence/ Worsening (43%)*
**De Freitas Dias, 2024 [20]**	Cohort	14	6	3M, 3F	5.5 (3-28)	Ataxia (4), Dystonia (1), Myoclonus (1)	Cortical lesions (4), Subcortical lesions (4), Brainstem (3)	Encephalopathy (5), Seizures (3), Cranial nerves (1), Motor symptoms (1), Bladder/Bowel symptoms (1)	Steroids (5), IVIG (6), PLEX (2)	Complete recovery
**George, 2024 [21]**	Retrospective Observational, Cohort design	61	7	N/A	7 (4-11)	Ataxia	Subcortical white matter, Thalamus, Brainstem, Cerebellum	Optic Neuritis, Headache, Altered Mental Status, Fever, Seizures	IV Steroids, IVIG<br><br>Maintenance: RTX, MMF	N/A
**Kim, 2024 [22]**	Retrospective Multicenter Cohort Study	235	7	N/A	N/A	Ataxia	Normal MRI (24%), Cortical Changes, Brainstem, Cerebellum, Leptomeningeal Enhancement	Headache, Seizures, Fever, Encephalopathy, Vomiting, Neuropsychiatric, Optic Neuritis, Spinal Cord Involvement	Steroids, IVIG<br><br>Maintenance: PLEX, MMF, AZA	N/A

**Wang, 2024 [23]**	Retrospective Observational Study	24	3	2M, 1F	4 (2.75-4.9)	Ataxia	Subcortical lesions (2), Brainstem (2), Cerebellum (2)	Fever (1)	Steroids (3), IVIG (3)	Partial Recovery
**Poovathingal, 2024 [24]**	Case series	8	5	3M, 2F	42 (3-73)	Ataxia (4), Parkinsonism (3), Tremor (4)	Subcortical lesions (2), Cerebellum (2), Spinal Cord (1)	Fever (1), Cranial nerves (1), Visual symptoms (1)	Steroids (5)<br><br>Maintenance: RTX (1)	Complete recovery (1), Improvement (4)
**Zhao, 2025 [25]**	Retrospective Observational, single center	11	11	N/A	3	Ataxia	Normal in 3 patients (rest N/A)	Encephalopathy, Headache, Vomiting	Immunotherapy (not specified)	2 patients relapsed

Abbreviations: MOGAD: Myelin Oligodendrocyte Glycoprotein Antibody-Associated Disease, IVIG: intravenous immunoglobulin, PLEX: Plasma Exchange, IFN: Interferon, MTX: Methotrexate, MiTX: Mitoxantrone, MMF: Mycophenolate Mofetil, AZA: Azathioprine, RTX: Rituximab, NTZ: Natalizumab, OCR: Ocrelizumab, IFX: Infliximab, LETM: Longitudinally Extensive Transverse Myelitis, N/A= not reported, regarding movement disorders, (n)=number of patients *percentages refer to the whole MOGAD cohort

Supplementary Table 1. Cohort and case-control studies included in the systematic review, describing MOGAD patients with movement disorders

1. Siritho S, Sato DK, Kaneko K, Fujihara K, Prayoonwiwat N: **The clinical spectrum associated with myelin oligodendrocyte glycoprotein antibodies (anti-MOG-Ab) in Thai patients**. *Mult Scler* 2016, **22**(7):964-968.
2. Jarius S, Kleiter I, Ruprecht K, Asgari N, Pitarokoili K, Borisow N, Hümmert MW, Trebst C, Pache F, Winkelmann A *et al*: **MOG-IgG in NMO and related disorders: a multicenter study of 50 patients. Part 3: Brainstem involvement - frequency, presentation and outcome**. *Journal of neuroinflammation* 2016, **13**(1):281.
3. Thulasirajah S, Pohl D, Davila-Acosta J, Venkateswaran S: **Myelin Oligodendrocyte Glycoprotein-Associated Pediatric Central Nervous System Demyelination: Clinical Course, Neuroimaging Findings, and Response to Therapy**. *Neuropediatrics* 2016, **47**(4):245-252.
4. Hacohen Y, Rossor T, Mankad K, Chong W, Lux A, Wassmer E, Lim M, Barkhof F, Ciccarelli O, Hemingway C: **'Leukodystrophy-like' phenotype in children with myelin oligodendrocyte glycoprotein antibody-associated disease**. *Dev Med Child Neurol* 2018, **60**(4):417-423.
5. Ramanathan S, O'Grady G L, Malone S, Spooner CG, Brown DA, Gill D, Brilot F, Dale RC: **Isolated seizures during the first episode of relapsing myelin oligodendrocyte glycoprotein antibody-associated demyelination in children**. *Dev Med Child Neurol* 2019, **61**(5):610-614.
6. Serin HM, Yilmaz S, Simsek E, Kanmaz S, Eraslan C, Aktan G, Tekgul H, Gokben S: **Clinical spectrum, treatment and outcome of myelin oligodendrocyte glycoprotein (MOG) antibody-associated disease in children: a tertiary care experience**. *Acta neurologica Belgica* 2021, **121**(1):231-239.

7. Alshamrani F, Alnajashi H, Shosha E, Casserly C, Morrow SA: **Case Series: Myelin Oligodendrocyte Glycoprotein-Immunoglobulin G-Related Disease Spectrum**. *Frontiers in neurology* 2020, **11**:89.
8. Banks SA, Morris PP, Chen JJ, Pittock SJ, Sechi E, Kunchok A, Tillema JM, Fryer JP, Weinshenker BG, Krecke KN *et al*: **Brainstem and cerebellar involvement in MOG-IgG-associated disorder versus aquaporin-4-IgG and MS**. *Journal of neurology, neurosurgery, and psychiatry* 2020.
9. Song X, Ma J: **Clinical characteristics of myelin-oligodendrocyte glycoprotein antibody-positive pediatric autoimmune encephalitis without demyelination: A case series**. *Frontiers in immunology* 2022, **13**:1050688.
10. Xiao J, Zhang SQ, Chen X, Tang Y, Chen M, Shang K, Deng G, Qin C, Tian DS: **Comparison of clinical and radiological characteristics in autoimmune GFAP astrocytopathy, MOGAD and AQP4-IgG(+) NMOSD mimicking intracranial infection as the initial manifestation**. *Multiple sclerosis and related disorders* 2022, **66**:104057.
11. Aubart M, Roux CJ, Durrleman C, Gins C, Hully M, Kossorotoff M, Gitiaux C, Levy R, Moulin F, Debray A *et al*: **Neuroinflammatory Disease following Severe Acute Respiratory Syndrome Coronavirus 2 Infection in Children**. *J Pediatr* 2022, **247**:22-28.e22.
12. Seneviratne SO, Marriott M, Ramanathan S, Yeh W, Brilot-Turville F, Butzkueven H, Monif M: **Failure of alemtuzumab therapy in three patients with MOG antibody associated disease**. *BMC neurology* 2022, **22**(1):84.
13. Boudjani H, Fadda G, Dufort G, Antel J, Giacomini P, Levesque-Roy M, Oskoui M, Duquette P, Prat A, Girard M *et al*: **Clinical course, imaging, and pathological features of 45 adult and pediatric cases of myelin oligodendrocyte glycoprotein antibody-associated disease**. *Multiple sclerosis and related disorders* 2023, **76**:104787.
14. Jiang Y, Tan C, Li X, Jiang L, Hong S, Yuan P, Zheng H, Fan X, Han W: **Clinical features of the first attack with leukodystrophy-like phenotype in children with myelin oligodendrocyte glycoprotein antibody-associated disorders**. *Int J Dev Neurosci* 2023, **83**(3):267-273.
15. Kang Q, Liao H, Yang L, Fang H, Ning Z, Liao C, Gan S, Wu L: **Clinical analysis of 173 pediatric patients with antibody-mediated autoimmune diseases of the central nervous system: a single-center cohort study**. *Frontiers in immunology* 2023, **14**:1140872.
16. Li L, Liu W, Cai Q, Liu Y, Hu W, Zuo Z, Ma Q, He S, Jin K: **Leptomeningeal enhancement of myelin oligodendrocyte glycoprotein antibody-associated encephalitis: uncovering novel markers on contrast-enhanced fluid-attenuated inversion recovery images**. *Frontiers in immunology* 2023, **14**:1152235.
17. Quack L, Glatter S, Wegener-Panzer A, Cleaveland R, Bertolini A, Endmayr V, Seidl R, Breu M, Wendel E, Schimmel M *et al*: **Autoantibody status, neuroradiological and clinical findings in children with acute cerebellitis**. *Eur J Paediatr Neurol* 2023, **47**:118-130.
18. Xu Q, Yang X, Qiu Z, Li D, Wang H, Ye H, Jiao L, Zhang J, Di L, Lei P *et al*: **Clinical features of MOGAD with brainstem involvement in the initial attack versus NMOSD and MS**. *Multiple sclerosis and related disorders* 2023, **77**:104797.
19. Abboud H, Sun R, Modak N, Elkasaby M, Wang A, Levy M: **Spinal movement disorders in NMOSD, MOGAD, and idiopathic transverse myelitis: a prospective observational study**. *Journal of neurology* 2024, **271**(9):5875-5885.
20. de Freitas Dias B, Toso FF, Barreto M, Dellavance A, Thomaz RB, Kowacs PA, Teive H, Spitz M, Juliano AFB, Rocha LJA *et al*: **Frequency of anti-MOG antibodies in serum and CSF of patients with possible autoimmune encephalitis: Results from a Brazilian multicentric study**. *Multiple sclerosis and related disorders* 2024, **92**:106171.
21. George E, Russ JB, Validrighi A, Early H, Mamlouk MD, Glenn OA, Francisco CM, Waubant E, Lindan C, Li Y: **Clinical and Imaging Findings in Children with Myelin Oligodendrocyte Glycoprotein Antibody Associated Disease (MOGAD): From Presentation to Relapse**. *AJNR American journal of neuroradiology* 2024, **45**(2):229-235.

22. Kim NN, Champsas D, Eyre M, Abdel-Mannan O, Lee V, Skippen A, Chitre MV, Forsyth R, Hemingway C, Kneen R *et al*: **Pediatric MOG-Ab-Associated Encephalitis: Supporting Early Recognition and Treatment**. *Neurol Neuroimmunol Neuroinflamm* 2024, **11**(6):e200323.
23. Wang Y, Guo X, Zhang L, Hua Y, Jing M, Hu X, Fan X, Sun M, Liu Y, Wang J: **Clinical characteristics analysis of 24 cases of pediatric MOG antibody-associated diseases**. *Multiple sclerosis and related disorders* 2024, **91**:105911.
24. Poovathingal MA: **The Varying Faces of MOGAD: A Case Series**. *Ann Afr Med* 2024, **24**(1):184-187.
25. Zhao Q, Gao C, Wang L, Liu C, Sun S, Li B: **Acute ataxia in children: etiological spectrum and clinical characteristics**. *Front Pediatr* 2025, **13**:1613558.
