# Supplementary material for: Movement Disorders in MOGAD: A Systematic Review
Source: Medicina (Kaunas). 2026 Apr 4;62(4):693. doi: 10.3390/medicina62040693 (PMC13117712; doi:10.3390/medicina62040693)
Supplement: Supplementary file 1 [file medicina-62-00693-s001.zip › Supplementary Table S2 SK final case reports.pdf]

Supplementary Table 2. Case reports of patients with MOGAD and movement disorders

Author, Year	Gender	Age (years)	Movement disorder	Movement disorder as presenting symptom (yes/no)	Imaging findings (lesions)	Other symptoms (during the same relapse)	Treatment (acute)	Treatment (Maintenance)	Outcome of movement disorder
**Mariotto, 2017 [1]**	M	31	Ataxia	Yes	Periventricular, thalamus, basal ganglia, internal capsule, mesial temporal lobes and brainstem	Encephalopathy, fever, headache	IVIG	no	Improvement
**Vieira, 2017 [2]**	F	9	Ataxia	Yes	Dorsal spinal cord at T2, T4-T5, and T6 levels, optic tracts, lateral geniculate nuclei	Encephalopathy, fever, headache, bilateral optic neuritis, bladder symptoms	Steroids (+tapering), IVIG	no	Complete recovery
**Gil Perotin, 2018 [3]**	M	46	Ataxia	No	Cortical, juxtacortical, periventricular, brainstem, cerebellum	Bilateral optic neuritis, dysphagia, dysarthria, left hypoesthesia, dysuria	No	MiTX, RTX	Deterioration
**Fujimori, 2019 [4]**	M	55	Ataxia	No	Right temporal lobe, left frontal and occipital lobe, left pons, left midbrain, left middle cerebellar peduncle.	Fever, Headache	Steroids (+tapering)	no	Complete recovery

**Sa, 2019 [5]**	F	3	Dystonia	Yes	Deep white matter (bilateral), brainstem and cortex	Encephalopathy, Fever, tetraparesis, focal seizure	Steroids (+tapering)	no	Complete recovery
**Yilmaz, 2019 [6]**	F	10	Ataxia	No	Basal ganglia (Gd enhancing), cerebral peduncles, cerebellum, cervical spinal cord	Encephalopathy	IVIG	no	Complete recovery
**Kumar, 2020 [7]**	F	37	Tremor	No	Cortical, bilateral thalami, brainstem, upper cervical cord extending to medulla	Vertigo, intractable vomiting, paraparesis, urinary and bowel retention.	IVIG, PLEX	no	Complete recovery
**El Jammal, 2021 [8]**	M	30	Ataxia	Yes	Pontomesencephalic and cerebellar lesions (Gd enhancing)	Paresis of the right arm	Steroids (+tapering)	no	Complete recovery
**Khan, 2021 [9]**	M	3	Dystonia	Yes	Bilateral insula and putamen, spinal cord T1/T2, T8 level	Encephalopathy, fever, seizures	Steroids (+tapering), IVIG, PLEX<br><br>diazepam, baclofen, and botulinum toxin injections	no	Improvement
**Krett, 2021 [10]**	M	69	Ataxia	Yes	Cervical spinal cord (Gd enhancing)	Headache, urinary hesitancy, constipation, hypopallesthesia in both legs	Steroids (+tapering)	AZA (after 2 relapses)	Improvement

**Adhikari, 2021 [11]**	F	26	Ataxia, Tremor, Myoclonus	Yes	Right periventricular and left globus pallidus	Vertigo	Steroids, IVIG	IVIG, RTX (after relapse)	Complete recovery
**Maniscalco, 2021 [12]**	F	28	Ataxia	No	Periventricular (enhancing), cerebellar peduncles bilaterally, brainstem	Dizziness, vomiting, hypoesthesia in the face	Steroids, PLEX	RTX, Steroids	Partial Improvement
**Smoot, 2021 [13]**	F	24	Ataxia	No	Pons, upper cervical cord (enhancing)	Diplopia, sensory disturbances in the upper extremities, nausea, constipation	Steroids (+tapering), IVIG	TCZ	Improvement
**Chakraborty, 2021 [14]**	M	10	Ataxia	Yes	Right midbrain and cerebellar hemisphere, bilateral periventricular	Dysarthria, strabismus	Steroids (+tapering)	MMF (after 3 relapses)	Complete recovery
**Bogdan, 2022 [15]**	M	47	Ataxia	Yes	Pons and medulla (Gd enhancing), periventricular	Tetraparesis	Steroids	MMF	Improvement
**Daems, 2022 [16]**	M	75	Ataxia	Yes	Pons-medulla, C3-C4	Micturition initiation problems, numbness of lower extremities, dysarthria, diplopia	Steroids, IVIG	No	Complete recovery

**Garcia Estrada, 2022 [17]**	F	2	Ataxia	Yes	Diffuse subcortical white matter, upper cerebellar peduncles, left cerebellar hemisphere	Behavioral changes	Steroids (+tapering)	RTX	Improvement
**Gibbons, 2022 [18]**	F	4	Ataxia	Yes	Both cerebellar hemispheres, cerebellar and cerebral peduncles, brainstem, thalami, left internal capsule	No	Steroids (+tapering)	No	Partial improvement
**Mayuzumi, 2022 [19]**	M	72	Ataxia	No	White matter, brainstem	Diplopia	Steroids	No	Improvement
**Redzek Mudrinic, 2022 [20]**	F	0.3	Dystonia	Yes	Thalamus, right lentiform nuclei, left internal capsule, right occipital cortex, left middle cerebellar peduncle, leptomeningeal enhancement	Encephalopathy, fever, tetraparesis	Steroids (+tapering)<br><br>Midazolam, phenobarbital for dystonic episodes	no	Complete recovery
**Zehden, 2022 [21]**	M	23	Ataxia	No	Basal ganglia, thalami	Encephalopathy, urinary retention, constipation, bilateral optic neuritis	Steroids	MMF (after 4 attacks)	Complete recovery
**Lopez, 2023 [22]**	M	3	Ataxia (1st attack)<br>Tremor (2nd attack)	Yes	white matter, thalami, brain penduncles, medulla to T10 (1st attack)	Tetraparesis (1st attack), encephalopathy (both attacks)	Steroids (+tapering)	no	Complete recovery

					cerebellar dentate nuclei (2nd attack)				
**Nakano, 2023 [23]**	F	23	Ataxia	No	Around fourth ventricle, right insular cortex and temporal pole (Gd enhancing)	Fever, headache, vertigo, vomiting	Steroids (+tapering)	no	Complete recovery
**Wang, 2023 [24]**	M	3	Ataxia	No	bilateral cerebellar hemispheres	No	Steroids (+tapering), IVIG	No	Improvement
**Xu, 2023 [25]**	F	31	Ataxia	No	Bilateral thalamus, basal ganglia, and right centrum semiovale (ADEM-like)	Paresthesias, blurry vision	Steroids (+tapering)	MMF	Complete recovery
**Holay, 2023 [26]**	N/A	20	Myoclonus	Yes	Right central sulcus oedema and leptomeningeal enhancement	Fever, headache, seizures, hemiparesis, aphasia	Steroids (+tapering), PLEX	steroids	Complete Recovery
**Okubo, 2024 [27]**	M	29	Ataxia	Yes	Spinal from C2 to conus, left pons	Fever, headache, sensory disturbance in lower extremities, urinary retention	Steroids (+tapering)	No	Complete recovery
**Schiro, 2024 [28]**	F	56	Ataxia	No	frontal, temporal, parietal white matter, cerebellum, midbrain, cerebellar peduncles, bulbar-	no	Steroids, PLEX	RTX, TCZ (after 3 relapses)	N/A

					medullary junction, T5				
**Zveik, 2024 [29]**	F	26	Tonic Spasms (dystonic episodes)	Yes	periventricular and subcortical lesions, corpus callosum, left centrum semiovale, corona radiata (enhancing)	N/A	Steroids (+tapering), IVIG, carbamazepine for tonic spasms	No	Complete recovery
**Mishan, 2025 [30]**	M	70	Tremor	Yes	Left optic nerve, T1-3, conus (enhancing)	Blurring of vision of left eye, urinary retention, left upper limb weakness	Steroids, PLEX	IVIG (after 1 relapse)	Improvement
**Baddam, 2025 [31]**	M	64	Ataxia	Yes	Periventricular, deep white matter, medulla (enhancing)	Encephalopathy, visual disturbance, nausea	Steroids (+tapering)	no	Improvement
**Sinha, 2025 [32]**	F	26	Ataxia	No	Bilateral subcortical frontoparietal, superior temporal, middle cerebral peduncles, cerebellar regions, thalami, and pons (enhancing)	Encephalopathy, headache	Steroids (+tapering)	no	Complete recovery

Abbreviations: Gd: gadolinium, IVIG: intravenous immunoglobulin, PLEX: Plasma Exchange, MiTX: Mitoxantrone, MMF: Mycophenolate Mofetil, AZA: Azathioprine, RTX: Rituximab, TCZ: Tocilizumab, N/A= not available

1. Mariotto S, Monaco S, Peschl P, Coledan I, Mazzi R, Höftberger R, Reindl M, Ferrari S: **MOG antibody seropositivity in a patient with encephalitis: beyond the classical syndrome**. *BMC neurology* 2017, **17**(1):190.
2. Vieira JP, Sequeira J, Brito MJ: **Postinfectious Anti-Myelin Oligodendrocyte Glycoprotein Antibody Positive Optic Neuritis and Myelitis**. *J Child Neurol* 2017, **32**(12):996-999.
3. Gil-Perotin S, Castillo-Villalba J, Carreres-Polo J, Navarré-Gimeno A, Mallada-Frechín J, Pérez-Miralles F, Gascón F, Alcalá-Vicente C, Cubas-Nuñez L, Casanova-Estruch B: **Progressive Demyelination in the Presence of Serum Myelin Oligodendrocyte Glycoprotein-IgG: A Case Report**. *Frontiers in neurology* 2018, **9**:340.
4. Fujimori J, Takahashi T, Matsumoto Y, Fujihara K, Takai Y, Misu T, Nakashima I: **Two Japanese cases of anti-MOG antibody-associated encephalitis that mimicked neuro-Behçet's disease**. *Journal of neuroimmunology* 2019, **334**:577002.
5. Sa M, Thornton R, Chong WK, Kaliakatsos M, Hacohen Y: **Paediatric MOG antibody-associated ADEM with complex movement disorder: A case report**. *Mult Scler* 2019, **25**(1):125-128.
6. Yılmaz Ü, Edizer S, Songür Ç Y, Güzin Y, Durak FS: **Atypical presentation of MOG-related disease: Slowly progressive behavioral and personality changes following a seizure**. *Multiple sclerosis and related disorders* 2019, **36**:101394.
7. Kumar N, Graven K, Joseph NI, Johnson J, Fulton S, Hostoffer R, Abboud H: **Case Report: Postvaccination Anti-Myelin Oligodendrocyte Glycoprotein Neuromyelitis Optica Spectrum Disorder: A Case Report and Literature Review of Postvaccination Demyelination**. *Int J MS Care* 2020, **22**(2):85-90.
8. El Jammal T, Jamilloux Y, Gerfaud-Valentin M, Richard-Colmant G, Weber E, Bert A, Androdias G, Sève P: **Challenging Mimickers in the Diagnosis of Sarcoidosis: A Case Study**. *Diagnostics (Basel)* 2021, **11**(7).
9. Khan TRW, JL; Wang, C.: **Anti-Myelin Oligodendrocyte Glycoprotein (MOG) antibody disease presenting with severe dystonia**. *Neuroimmunology Reports* 2021, **1**.
10. Krett JD, Jarvis SE, Alikhani K: **Myelin Oligodendrocyte Glycoprotein Antibody-Associated Myelitis Presenting with Headache**. *The Canadian journal of neurological sciences Le journal canadien des sciences neurologiques* 2021, **48**(6):879-881.
11. Adhikari S, Thuringer A, Maali L, Jassam Y: **Opsoclonus myoclonus syndrome in a postpartum period**. *Multiple sclerosis and related disorders* 2021, **50**:102862.

12. Maniscalco GT, Allegorico L, Alfieri G, Napolitano M, Ranieri A, Renna R, Servillo G, Pezzella M, Capone E, Altomare L *et al*: **Anti-MOG-associated demyelinating disorders: two sides of the same coin**. *Neurological sciences : official journal of the Italian Neurological Society and of the Italian Society of Clinical Neurophysiology* 2021, **42**(4):1531-1534.
13. Smoot KCC, RN; Cohan, S. : **Recurrent relapse after 20 years in a patient with MOG antibody disease: A case report**. 2021, **1**.
14. Chakraborty U, Ghosh S, Datta AK, Chandra A: **Recurrent ataxia and dysarthria in myelin oligodendrocyte glycoprotein antibody-associated disorder**. *BMJ case reports* 2021, **14**(11).
15. Bogdan T, El Ghannudi S, Demuth S, Kremer L, De Seze J, Bigaut K: **Reverse Takotsubo cardiomyopathy as a complication of MOG-antibody-associated disease (MOGAD)? A case report**. *Revue neurologique* 2022, **178**(9):981-983.
16. Daems F, Derdelinckx J, Ceyssens S, Vanden Bossche S, Reynders T, Willekens B: **Improved detection of MOG antibody-associated transverse myelitis with 18F-FDG-PET: a case report**. *Acta neurologica Belgica* 2023, **123**(2):735-738.
17. García-Estrada CG-F, E; Morelos-Cisneros, JP; Deras-Martinez A.: **Myelin oligodendrocyte glycoprotein antibody-associated disease presenting as recurrent acute disseminated encephalomyelitis: Case report of the youngest Mexican**

patient in the literature. *Clinical and Experimental Neuroimmunology* 2022, **13**(4):256-259.
18. Gibbons E, Whittam D, Elhadd K, Bhojak M, Rathi N, Avula S, Jacob A, Griffiths M, Huda S: **Progressive myelin oligodendrocyte glycoprotein-associated demyelination mimicking leukodystrophy**. *Mult Scler* 2022, **28**(9):1481-1484.
19. Mayuzumi Y, Kitazawa Y, Kunimatsu T: **Relapse of myelin oligodendrocyte glycoprotein antibody-associated demyelinating disease in an elderly patient after long-term remission**. *Acta neurologica Belgica* 2023, **123**(4):1531-1532.
20. Redžek-Mudrinić TK, I.; Koprivšek, K.; Rakić, G.; Pajić, J.: **Pediatric acute disseminated encephalomyelitis associated with myelin oligodendrocyte glycoprotein antibodies**. *Srp Arh Celok Lek* 2022, **150 (3-4)**:212-215.
21. Zehden J, Harish Bindiganavile S, Bhat N, Lee AG, Avery R, Golnik KC: **Delayed Diagnosis of Anti-Myelin Oligodendrocyte Glycoprotein One Decade After Presumed Recurrent Acute Disseminated Encephalomyelitis**. *J Neuroophthalmol* 2022, **42**(2):e469-e472.
22. Lopes TC, C.; Gonçalves, R.; Pais, RP; Palavra; F.: **Atypical Cerebellar Involvement in MOG Antibody-Associated Disease (MOGAD) in Early Childhood**. *Sinapse* 2023, **23**:60-61.
23. Nakano H, Yamaguchi K, Hama N, Matsumoto Y, Shinohara M, Ide H: **Relapsing Anti-MOG Antibody-associated Disease following COVID-19 Vaccination: A Rare Case Report and Review of the Literature**. *Internal medicine (Tokyo, Japan)* 2023, **62**(6):923-928.
24. Wang X, Zhao R, Yang H, Liu C, Zhao Q: **Two rare cases of myelin oligodendrocyte glycoprotein antibody-associated disorder in children with leukodystrophy-like imaging findings**. *BMC neurology* 2023, **23**(1):247.
25. Xu M, Ma C, Dong M, Guo C, Yang S, Liu Y, Wang X: **Two case reports and a systematic review of the literature on adult cerebral cortical encephalitis with anti-myelin oligodendrocyte glycoprotein antibody**. *Frontiers in immunology* 2023, **14**:1203615.

26. Holay Q, Gazzola S, Quesnel L, Faivre A: **Migrating cortical lesion in FLAIR-hyperintense lesions in anti-MOG-associated encephalitis with seizures**. *Journal of neurology, neurosurgery, and psychiatry* 2023, **94**(10):871-872.
27. Okubo S, Kakumoto T, Tsujita M, Muramatsu K, Fujiwara S, Hamada M, Satake W, Toda T: **Extremely Longitudinally Extensive Transverse Myelitis in a Patient With Myelin Oligodendrocyte Glycoprotein Antibody-Associated Disease**. *Cureus* 2024, **16**(5):e59938.
28. Schirò G, Iacono S, Andolina M, Bianchi A, Ragonese P, Salemi G: **Tocilizumab treatment in MOGAD: a case report and literature review**. *Neurological sciences : official journal of the Italian Neurological Society and of the Italian Society of Clinical Neurophysiology* 2024, **45**(4):1429-1436.
29. Zveik OBB, T.; Keadan, T.; Barhum, Khalil; Rechtman, Ariel; Vaknin-Dembinsky, A. : **Myelin oligodendrocyte glycoprotein antibody-associated disease presenting with dystonia**. *Neurology and Clinical Neuroscience* 2023, **12**(1):65-76.
30. Mishan YS, D.; Elefant, D.; Gandelman, S. : **Late-onset MOGAD: A case series and literature review**. 2025, **8**.
31. Baddam S, Patel S, Kahlon N, Thiriveedi M: **Unmasking Myelin Oligodendrocyte Glycoprotein Antibody-Associated Disease (MOGAD): CNS Demyelination Triggered by TNF-α Inhibition in a Patient with Ankylosing Spondylitis**. *Eur J Case Rep Intern Med* 2025, **12**(6):005467.
32. Sinha NP, P.; Ranjan, A.: **Chronic progressive behavioral changes associated with headache: An atypical presentation of myelin oligodendrocyte glycoprotein (MOG)-associated disease**. 2025, **31**(1).
